# The Role of Stakeholders’ Understandings in Emerging Antimicrobial Resistance: A One Health Approach

**DOI:** 10.3390/microorganisms11112797

**Published:** 2023-11-17

**Authors:** Patrizia Nardulli, Andrea Ballini, Maria Zamparella, Danila De Vito

**Affiliations:** 1 S.C. Farmacia e UMACA IRCCS Istituto Tumori “Giovanni Paolo II”, Viale O. Flacco 65, 70124 Bari, Italy; p.nardulli@oncologico.bari.it; 2 Department of Clinical and Experimental Medicine, University of Foggia, 71122 Foggia, Italy; 3 Local Health Authority ASL/BA, 70125 Bari, Italy; zamparella.maria@gmail.com; 4 Department of Translational Biomedicine and Neuroscience, Medical School, University Aldo Moro of Bari, 70124 Bari, Italy; danila.devito@uniba.it

**Keywords:** multi-resistant strains, antibiotics, environmental impact, veterinary medicine

## Abstract

The increasing misuse of antibiotics in human and veterinary medicine and in agroecosystems and the consequent selective pressure of resistant strains lead to multidrug resistance (AMR), an expanding global phenomenon. Indeed, this phenomenon represents a major public health target with significant clinical implications related to increased morbidity and mortality and prolonged hospital stays. The current presence of microorganisms multi-resistant to antibiotics isolated in patients is a problem because of the additional burden of disease it places on the most fragile patients and the difficulty of finding effective therapies. In recent decades, international organizations like the World Health Organization (WHO) and the European Centre for Disease Prevention and Control (ECDC) have played significant roles in addressing the issue of AMR. The ECDC estimates that in the European Union alone, antibiotic resistance causes 33,000 deaths and approximately 880,000 cases of disability each year. The epidemiological impact of AMR inevitably also has direct economic consequences related not only to the loss of life but also to a reduction in the number of days worked, increased use of healthcare resources for diagnostic procedures and the use of second-line antibiotics when available. In 2015, the WHO, recognising AMR as a complex problem that can only be addressed by coordinated multi-sectoral interventions, promoted the One Health approach that considers human, animal, and environmental health in an integrated manner. In this review, the authors try to address why a collaboration of all stakeholders involved in AMR growth and management is necessary in order to achieve optimal health for people, animals, plants, and the environment, highlighting that AMR is a growing threat to human and animal health, food safety and security, economic prosperity, and ecosystems worldwide.

## 1. Introduction

Bacterial infections have impacted humans throughout the centuries, and were particularly harmful until the discovery of antibiotics, which revolutionized the treatment of infectious diseases. Because of their ability to survive in different environments, bacteria are increasingly able to face antibacterial treatments over time by means of different adaptative strategies [1].

Antimicrobial resistance (AMR) threatens one of the greatest discoveries of modern medicine, antibiotics, compromising their effectiveness in the fight against pathogenic microorganisms [1,2]. Without global action, we risk entering a new post-antibiotic era in which trivial infections can, as in the past, kill [3,4,5,6]. In 2015, the WHO recognised AMR as a complex problem that can only be addressed via coordinated multi-sectoral interventions, promoting the One Health approach that considers human, animal and environmental health in an integrated way (WHO, 2015), and in 2017, a One Health Action Plan was developed involving European Member States in strong international cooperation [2]. The Food and Agriculture Organization of the United Nations (FAO), the UN Environment Programme (UNEP), the WHO, and the World Organisation for Animal Health (WOAH) have launched the Antimicrobial Resistance Multi-Stakeholder Partnership Platform to address the growing threats and impacts of AMR globally. The platform aims to mobilize coordinated efforts by a large number of stakeholders and bring together voices from all areas, sectors, and perspectives through a holistic and system-wide One Health approach [6].

The goals were the following:

1. Preserve the possibility of effective treatment of infections in both humans and animals [7,8,9];
2. Implement legislation for monitoring AMR in zoonotic and commensal bacteria in both animals and food [10,11];
3. Stimulate the creation of a network of linked laboratories in networking to improve and monitor the isolation of multi-resistant microorganisms in humans [12,13,14,15];
4. In cooperation with European Food Safety Agency (EFSA), stimulate the identification of resistant bacteria responsible for transmissible diseases in animals [16] and to this end initiate surveillance programmes;
5. Support good practice in the control and prevention of hospital-associated infections [17,18];
6. Support research in the development of new antibiotic molecules within and outside the EU [19,20].

It is therefore logical to adopt a “One Health” approach to address this problem. AMR is one of the major health problems we face in the 21st century. Nowadays, we cannot understand global health without the interdependence between the human, animal, and environmental aspects, taking also into account the cross-talk with the agroecosystem dimensions. In this review, we try to address why a collaboration of all stakeholders involved in AMR growth and management is necessary in order to achieve optimal health for people, animals, plants, and our environment. 

## 2. Antibiotic Resistance in the Veterinary Sector

Antibiotics have played an essential role in the treatment of numerous infectious diseases, contributing to a significant improvement in the health status of animals as well, and speeding up their growth processes through the control of food-related infections [21,22,23]. This improvement risks being undermined by the increasing spread of pathogens resistant to antibiotics [24,25,26]. The Antimicrobial Resistance Plan 2017–2020 reports that more than 50% of antibiotics used globally are consumed in veterinary medicine [27,28]. The incorrect use of antibiotics in veterinary medicine also increases selective pressure and the spread of resistant bacteria, which can be transferred from animals to humans [27,28,29] either by direct contact or through food of animal origin, or indirectly through more complex cycles of environmental contamination [30].

Indeed, the use of antibiotics in animal husbandry can disrupt the delicate balance of the intestinal microbiome, a microbial community that plays a crucial role in maintaining the health of both humans and animals [31,32]. The intricate connections between humans, animals, and the environment are evident in the impact that antibiotic use in animals can have on the microbiome, as it can potentially lead to imbalances with far-reaching consequences. Understanding and addressing this interference is important, not only for the well-being of animals, but also for the broader context of public health and environmental sustainability [33,34,35].

Also, as aspect not to be underestimated is an increase in the potential health risk for farmers, which may be responsible for a reduction in both farm efficiency and production safety.

The use of antibiotics in animal husbandry presents a multifaceted set of risks and concerns. On one hand, it is well-established that there are risks of environmental contamination due to the presence of antibiotic-resistant bacteria in the waste products of treated animals [36]. Additionally, there is a direct risk for veterinarians, breeders, and workers who may acquire antibiotic resistance through prolonged or repeated exposure to these drugs [37,38,39].

However, the impact of antimicrobial use in animal husbandry on the risk of transmitting antibiotic-resistant bacteria to humans, particularly through the consumption of animal-derived food products, remains an area that requires further investigation [35,36,37,38,39]. This is a critical issue as it relates to food safety and public health, and ongoing research is needed to better understand the extent of this risk and how it can be effectively managed. The complex interplay of antibiotic use, resistance development, and transmission to humans, underscores the importance of a comprehensive and multidisciplinary approach to address this issue [40,41].

The prudent use of antibiotics, moreover, can only be closely linked to the application of high standards of farm welfare and biosecurity [36,37,42]. It follows that an integrated approach to the phenomenon of antibiotic resistance is a key element in combating its occurrence [38,39,41]. Key actions in veterinary medicine include a computerised system of traceability of veterinary medicines, including the Electronic Veterinary Recipe, compulsory from 16 April 2019; a drug classification system for risk categorisation within herds; good pharmaco-surveillance; appropriate training of veterinarians, pharmacists, and operators; guidelines for the prudent use of antibiotics; funding of research on antibiotic resistance in the veterinary field; participation in the European surveillance system on the sale of antimicrobials in the veterinary sector (ESVAC) project. The European Centre for Disease Prevention and Control (ECDC) states in one of its latest reports that the veterinary sector in Italy has significantly reduced the use of antibacterial drugs in the livestock sector since 2014, showing an even lower overall use of antibiotics per kg of biomass than in the human sector. The improvement described so far has also been achieved thanks to the electronic prescription system that started in Italy and Spain even two years before the entry into force of the aforementioned new European regulation, which was also adopted in response to antibiotic sales that were too high compared to those of other Member States [43]. The European Medicines Agency (EMA) has issued a scientific opinion on the designation of antimicrobials to be reserved for the treatment of certain human infections, to preserve their efficacy and reduce the risk of antibiotic resistance to public health [44,45].

## 3. Antibiotic Resistance in the Environmental Sector

The misuse of antibiotics in human and veterinary medicine has led to the development and proliferation of specific resistances in bacterial communities exposed to the effects of human activities all over the planet [6,26]. The abundance and diversity of resistance genes and resistant bacteria in the environment are closely related to the impact caused locally by human activities [19,43]. Antibiotic resistance resulting from resistance genes to synthetic and semi-synthetic antibiotics [13,23,39] spreads in the environment via multiple contamination pathways as a result of different anthropogenic activities [46,47,48], in which there is a high use of antibiotics [41]. Resistance genes can reach the environment both through diffuse sources of contamination [49], i.e., areas of intensive agriculture [50,51], industrial districts, and human activities distributed throughout the territory, and through point sources, such as intensive livestock facilities [46], aquaculture, urban [52,53] and hospital sewage discharges [47,50], and industrial activities for the production of antibiotic substances. 

Antibiotic use in animals can impact human health in different ways, each presenting its own set of complex challenges and considerations. These multifaceted effects need to be thoroughly understood and addressed to develop effective policies and practices related to antibiotic use in agriculture. The impact of the massive use of antibiotics causes not only the release of resistant bacteria and resistance genes into the environment, but also significant quantities of the various antibiotics [54]. The parent substances not metabolised by the human body and their metabolites are where they are generally not completely removed. Antibiotics and metabolites are then released into streams, lakes, or the sea [55] through treated water or into the soil through the use of sewage sludge as fertiliser for fields [56]. This class of contaminants, despite its heterogeneity, is generally referred to as ‘semi-persistent’ because its use is continuous and massive: significant quantities are released into the environment on a daily basis as a result of use in human and veterinary medicine. In practice, although some substances degrade rapidly in the environment, they are always present due to continuous input. Prevention activities at the source of the discharge and disposal of antibiotic substances into the environment represent the priority strategy for action. It is important to strengthen environmental and urban wastewater monitoring networks to support knowledge-based intervention measures and best available techniques. In Italy, the Ministry of Ecological Transition, in collaboration with researchers from the Italian National Institute for Environmental Protection and Research (ISPRA), the National Research Council, universities, and scientific research institutes, has proposed a number of priority actions to facilitate the proper management of antibiotic resistance in the environment [57,58,59]; review the management of antibiotics and waste in intensive livestock farms [23]; convey, through the tools of information and education of the population, indications on the correct use and disposal of antibiotic drugs [58]; and promote research activities on the relationship between antibiotic resistance and the environment [60].

Furthermore, the role of degradable plastics should not be underestimated, which are not fully biodegradable and are increasing in use. What is relevant is the problem of microplastic pollution (MPs), one of the main environmental issues of the last decade. MPs are defined as particles of anthropogenic origin between 100 nm and 1 mm, if small, and between 1 and 5 mm, if medium-sized. MPs are purposely added in several products to change their consistency, stability or to impart functions, such as abrasive capacity; for these purposes, they are added, for example, in fertilisers and plant protection products, industrial and household detergents, paints, and products used in the oil and gas industry [61,62,63,64,65,66,67]. MPs persist in the environment in large quantities, especially in marine and aquatic ecosystems, and it is estimated that in the oceans, more than 68% of MPs derive from undisposed or improperly disposed of and released into the environment.

All this contributes to the permanent pollution of ecosystems and their accumulation along the food chain, with both direct and indirect negative effects on human health. Human beings may, in fact, suffer a physical reaction, linked to the size of plastic fragments, a chemical reaction, due to the possible release of monomers, additives and chemical agents, and also due to the deterioration of plastic fragments, and finally, a biological reaction, due to the colonisation of MPs by pathogenic microorganisms [60]. The compounds that most commonly form MPs are compounds such as polyethylene and polyvinyl chloride, materials that due to their chemical–physical properties facilitate the binding and transport of chemical contaminants, such as antibiotics, and microbial agents [68,69,70,71,72,73,74,75], such as bacteria, increasing their impact on the environment and human health [76,77,78,79,80,81,82].

Some studies on plastisphere, examined the role of polystyrene take-away food containers in the formation of MPs and their potential contribution to the growing problem of antibiotic resistance [80,81]. Once polystyrene is transformed into MPs, it can serve as an ideal substrate for hosting microorganisms and chemical contaminants. Moreover, it can harbour genetic materials containing antibiotic resistance genes (ARGs) [82,83]. These studies highlighted how the aging of these MPs in the environment makes them particularly conducive to binding with ARGs [82,83].

The aging process of MPs can be triggered by factors such as mechanical abrasion, exposure to solar radiation, and biodegradation. These processes increase the surface area of MPs, break down their polymer structure, and alter their physical and chemical properties. This, in turn, promotes microbial adhesion and the formation of biofilms.

Additionally, the widespread and often inappropriate use of antibiotics in various human activities leads to their release into the environment, primarily through wastewater. In such environments, ARGs can transfer to pathogenic bacteria via horizontal gene transfer methods like conjugation, transformation, or transduction [64,65,66,67].

The release of depolymerizing chemicals from MPs can change the membrane permeability of microorganisms, potentially facilitating the transfer of ARGs. Additives or pollutants accumulated on MPs can also influence the presence of ARGs. For example, the presence of copper and zinc on plastic surfaces can promote the binding of ARGs related to resistance against certain antibiotics. Organic pollutants like polycyclic aromatic hydrocarbons have been found to exert selective pressure on the transfer of ARGs through mechanisms like co-selection or cross-selection [82,84].

Specifically, in 2023, the study of Tuvo et al. [82] reveals that the presence of copper sulphate resistance genes can co-select resistance to various antibiotics that share the same genetic element. MPs have the capacity to transport or facilitate the exchange of ARGs and other pollutants across different environmental compartments, even over long distances.

In conclusion, the presence of ARGs on MPs dispersed in the environment presents an emerging concern that will need to be addressed in the near future through proper management of plastic waste disposal and effective water treatment methods. This is especially critical in the case of hospital wastewater, where the concentration of ARGs and the potential for antibiotic resistance spread is particularly high.

It is therefore essential to deepen our knowledge of the environmental behaviour of MPs and their role in the transmission of ARGs by studying the MPs resistome and how much of this is shared with the surrounding environment [23,24], to understand the potential risk of exposure for humans [83,84,85,86,87]. The importance of the environment and food of both animal and plant origin in the emergence of emerging diseases, such as SARS and avian influenza, allowed the term ‘One Health’ to be coined in 2003 and laid the foundations for the importance of collaboration between different professions to respond with a strategic approach to prevention and surveillance of emerging diseases [3,8,70].

## 4. Antibiotic Resistance and Impact on Humans

Bacterial infections plagued human societies throughout history until the discovery of antibiotics [72,73,74,75,76], which led to adapt in different environments, the threat of antibiotics by means of different astute strategies [4,6,13,73]. They can modify the quaternary structure of a target protein, substitute a metabolic pathway by synthesizing alternative biomolecules, expel antibiotics from their cell by efflux pumps, produce enzymes able to inactivate the antibiotic, hide their target structure, for example, behind an outer capsule [76,87,88,89]. Antimicrobial resistance genes may be carried on the bacterial gene chromosome, plasmid, or transposons [90,91,92,93,94,95].

Bacteria are also capable of forming biofilms that physically prevent host immune response cells and antibiotics from inhibiting the pathogen. In addition, biofilms protect the dormant cells called persistent cells that are an expression of antibiotic tolerance, in which case the microorganisms are resistant in vivo to high doses of antibiotics without manifesting resistance to the minimum inhibitory concentration (MICs), in vitro [60,62]. Antibiotic tolerance can arise when bacteria are exposed to environmental conditions of stress, temperature, reduced nutrient supply and treatment with antibiotics [96,97,98,99,100,101]. A previous study in 2016 conducted by de Kraker et al. [97] on antimicrobial resistance estimated that worldwide, by 2050, bacterial infections will cause around 10 million deaths per year, far exceeding deaths from cancer (8.2 million), diabetes (1.5 million) or traffic accidents (1.2 million) with a projected cost for “Combatting Bacterial Resistance in Europe: COMBACTE-NET” exceeding euro 220,000,000.00.

The WHO List of Critically Important Antimicrobials for Human Medicine (WHO CIA List) was initially established based on the recommendations from two successive expert meetings jointly organized by the Food and Agriculture Organization of the United Nations (FAO), the World Organization for Animal Health (OIE), and the World Health Organization (WHO).

During the first expert workshop, it was concluded that there was compelling evidence of adverse impacts on human health due to the development of antimicrobial resistance in non-human usage of antimicrobials. This included an increased frequency of infections, higher rates of treatment failures (sometimes resulting in fatalities), and greater severity of infections. These consequences were well-documented, particularly in cases of fluoroquinolone-resistant human Salmonella infections [101].

In 2008, the American Society of Infectious Diseases coined the acronym ESKAPE. To date, ESKAPE pathogens are responsible for the majority of hospital infections and owe their name to their ability to escape (from the English ‘to escape’) the biocidal action of antimicrobial agents [102,103]. The acronym ESKAPE includes the six nosocomial pathogens that show multi-resistance and virulence: *Enterococcus faecium*, *Staphylococcus aureus*, *Klebsiella pneumoniae*, *Acinetobacter baumannii*, *Pseudomonas aeruginosa*, and *Enterobacter* Species. ESKAPE pathogens are responsible for the majority of nosocomial infections and are able to ‘escape’ the biocidal action of antimicrobial agents. They are also associated with the highest risk of mortality, leading to increased healthcare costs [104,105,106,107]. The World Health Organisation also recently included ESKAPE pathogens in its list of 12 bacteria against which new antibiotics are urgently needed [105]. Three categories of pathogens are described: critical, high, and medium priority, depending on the urgency of the need for new antibiotics. Carbapenemase-resistant *A. baumamannii* and *Pseudomonas aeruginosa* [94,95] together with extended-spectrum β-lactamase (ESBL) or carbapenemase-resistant *K. pneumoniae* and *Enterobacter* spp. [108,109] are listed in the critical priority list of pathogens; whereas vancomycin-resistant *E. faecium* (VRE) and methicillin- and vancomycin-resistant *S. aureus* (MRSA AND VRSA) are in the high priority group list [110,111,112,113,114,115].

According to the European Centre for Disease Prevention and Control (ECDC), the most common and clinically relevant bacterial species in European hospital isolates include *Escherichia coli*, *Pseudomonas Aeruginosa*, *Klebsiella Pneumoniae*, *Staphylococcus Aureus*, and *Enterococcus Faecium* [116,117,118,119,120,121].

Among hospital-acquired (HA) infections in Europe, 41% of *S. aureus* infections are methicillin-resistant, and particularly in Northern Europe (i.e., the United Kingdom and France), a steady decrease in the prevalence of HA-MRSA was observed between 2015 and 2018 and was largely attributed to improved national infection control programs. In comparison, the rates of HA-MRSA in Southern Europe (i.e., Portugal, Spain, Italy, and Greece) remain high [117,118,119,120,121,122,123,124,125,126].

In Europe 32% of *P. aeruginosa* infections are resistant to carbapenems (ESBL-carba) [119]. Furthermore, the bacteria remain a major contributor of hospital-acquired infection [120,121]. Widespread distribution of *P. aeruginosa* nosocomial isolates resistant to last-resort and polymyxin- and carbapenem-class antibiotics is well documented [115,127].

With regard to *Klebsiella pneumoniae*, the Italian 2019 statistics report that 30% of the strains isolated are multi-drug-resistant (MDR), and in particular, between 2005 and 2010, we showed an increase in isolates causing invasive infections [115,128,129,130,131,132,133].

Twelve countries reported rates of 25% or more, six of which reported AMR rates of 50% or more (Belarus, Georgia, Greece, Republic of Moldova, Russian Federation, and Ukraine) [116,134,135,136,137,138].

## 5. Antibiotic Resistance in Agroecosystems

Currently, communication across the One Health triad (humans, animals, environment) regarding agricultural AMR is hindered by ambiguous language, complicated by cultural and linguistic differences that can lead to the conclusion that the other participant is not aware of the facts or has ulterior motives [139,140].

Antibiotic use in agriculture is just one factor contributing to the rise in antimicrobial resistance. Modern factory farms are the ideal breeding grounds for antibiotic-resistant infections: many animals are raised in crowded, unsanitary conditions. The debate regarding the impact of antibiotic use in agriculture on the development of clinically relevant antibiotic resistance in human medicine is fuelled and perpetuated by the challenge of obtaining direct, quantitative data to assess the scale and nature of this contribution [139].

In fact, a substantial portion of antibiotics produced in the United States, are used in agricultural practices [10]. This use has undoubtedly played a role in the prevalence of antibiotic-resistant bacteria in the gut flora of food animals like chickens and swine. However, regulating the use of antibiotics in agriculture has been a contentious issue. Policymakers have been tasked with balancing the evident benefits of antibiotic use for animal health and economic advantages for food producers, pharmaceutical companies, and potentially consumers, against the nebulous threat to human health that is often challenging to precisely quantify [10]. 

It is important to note that antibiotic drugs and their bioactive breakdown products, while not technically classified as having “antibiotic resistance” characteristics themselves, are widely recognized as the primary driver of antibiotic resistance [141]. The presence of these drugs and their metabolites in the environment is a significant concern. Discussions aimed at addressing agricultural and environmental antibiotic resistance often revolve around the impact of anthropogenically controlled drugs in various environmental matrices.

The release of antibiotics into the environment, whether through agricultural use, wastewater discharges, or other means, can contribute to the selection and proliferation of antibiotic-resistant bacteria [142]. These resistant bacteria can, in turn, pose a risk to human and animal health as they may enter the food chain, spread in the environment, and potentially transfer resistance genes to other bacteria.

Efforts to address AMR must consider the environmental dimension and the role of antibiotics in driving resistance. It is essential to explore strategies for responsible antibiotic use in agriculture, effective wastewater treatment, and measures to mitigate the environmental impact of these drugs to help combat the growing challenge of AMR.

As an example, significant is the use of ionophores in agriculture, with over 4 million kilograms sold in the United States in 2016 [143,144,145]. Even if on one hand, ionophores are the second most widely used class of antibiotics in agriculture, on the other hand are not used in human medicine, there has been an assumption that their use in agriculture does not directly impact human health, leading in this way that have not been subjected to the same regulatory scrutiny as medically important antibiotics [144,145]. 

However, there is a growing concern that the current evidence base is insufficient to definitively conclude that ionophores do not contribute to antimicrobial resistance relevant to human health. Several factors contribute to this uncertainty:

(1) Cross-Resistance: It is unclear whether resistance to ionophores in bacteria can lead to cross-resistance to medically important antibiotics. This is a significant concern, as cross-resistance could undermine the effectiveness of crucial human antibiotics [145,146].
(2) Co-selection: Recent evidence suggests that the use of ionophores may have the unintended consequence of co-selecting for resistance to antibiotics used in human medicine. For example, there are indications that ionophore use might contribute to vancomycin resistance in some cases [145].

Given these concerns, there is a need for further research and investigation into the potential risks associated with ionophore use in agriculture. The goal is to better understand the implications of antibiotics, such as ionophore resistance and their potential impacts on human health. This ongoing examination is essential to inform responsible and evidence-based regulations and practices in the use of antibiotics in agriculture, ensuring the protection of both animal and human health.

## 6. Therapeutic Advances against ESKAPE Pathogens

Today, for ESKAPE pathogens, there are few antimicrobial products on the market [128]. The first antibiotic, penicillin, dates back to 1928, when Alexander Fleming noticed the antibacterial activity of a mould (*Penicillium notatum*) against colonies of *Staphylococcus aureus*. *S. aureus* first showed resistance to methicillin in 1961, as a consequence of the widespread use of penicillin. The introduction of penicillin also increased the emergence of penicillinase-producing *S. aureus.* Since the introduction of the first drugs with antibiotic activity in the 1940s and with their subsequent spread worldwide, antibiotics have saved hundreds of millions of lives (World Bank, 2016, [130]). Since the early 1960s, only four new classes of antibiotics have been introduced: quinolones, lincosamides, oxazolidinones, and cyclic lipopeptides [96] (as shown in Table 1).

The acronym AWaRE stands for Access, Watch and Reserve, which are the categories in which antibiotics are classified in the WHO model list of essential medicines [131]. In 2019, the WHO launched the AWaRe campaign to promote the use of a tool to help governments curb the growth of antibiotic resistance and make the use of antibiotics safer and more effective, reducing costs and the occurrence of adverse events.

The ‘Access’ category includes the antibiotics of choice for the 25 most common infections.

The use of ‘Access’ antibiotics reduces the risk of resistance because they have a limited spectrum.

The ‘Watch’ category includes most of the important antibiotics of high priority and criticality, for use in both human and veterinary medicine. These antibiotics are only recommended for specific and limited indications.

Antibiotics in the ‘Reserve’ category should be used as a last resort, when all other antibiotics have failed [132].

According to the WHO General Programme of Work 2019–2023, in order to optimise the use of antibiotics and reduce resistance, it is necessary to increase the proportion of ‘Access’ antibiotics to at least 60% of national consumption and reduce the use of those at greater risk of developing resistance in the ‘Watch’ and ‘Reserve’ categories. Italy is among the European countries with the lowest consumption of antibiotics in the Access group, i.e., those of first choice, while it has a higher incidence of drugs in the Watch group, antibiotics that should be used with caution because of the greater risk of inducing resistance. External pressures from governments and consumers play a significant role in influencing farming practices, particularly in the context of responsible animal welfare and antibiotic use.

Alternative therapies to the use of antibiotics include bacteriophage therapy, probiotics [133,134,135], drug repurposing [136], monoclonal antibody (MAb), and (v) faecal microbiota transplantation (FMT) [137], which are some of the many novel approaches currently being investigated to control the AMR. Balancing these factors can be challenging, and it requires collaboration between the agricultural industry, consumers, and policymakers [138]. This shift towards more responsible practices is a part of a larger global effort to ensure a safer, more ethical, and sustainable food supply chain.

## 7. Future Perspectives

Antibiotic resistance is a phenomenon that affects all countries in the world and is burdened by high mortality in countries (South East Asia, Africa) that have not yet undertaken surveillance and prevention programmes [147,148]. The phenomenon was exacerbated by the acquisition by Gram-positive and Gram-negative bacteria of resistance genes, which made them able to escape the biocidal action of antibiotics, AMR ESKAPE pathogens [148]. Hospital infections caused by these micro-organisms are burdened with increased costs for prolonged hospitalisation and treatment and correlate with increased hospital mortality rates when the bacteria cause sepsis [147].

Addressing antimicrobial resistance in livestock farming is indeed a complex challenge. The decisions regarding antibiotic use should ideally prioritize the welfare of the animals while also minimizing the risk of antimicrobial resistance. This involves several key principles:

(1) Avoiding Prophylactic Use: Antibiotics should not be used routinely or prophylactically but rather in response to identified infections or under the guidance of a veterinarian.
(2) Choosing the Right Antibiotic: When antibiotics are necessary, selecting the correct antibiotic is essential. This should be based on diagnostic tests and knowledge of the specific bacteria causing the infection.
(3) Correct Dosage and Duration: Using the right dose for the right duration is critical to ensure the infection is properly treated, reducing the likelihood of resistance development.
(4) Prevention: The most effective strategy is to prevent infections from occurring in the first place. This includes maintaining good hygiene, implementing biosecurity measures, and managing animal health and living conditions.

The agricultural sector plays a crucial role in the One Health framework, and the sector’s active involvement is vital in the global effort to address AMR comprehensively and sustainably. In addition, involving agricultural experts in the conversation and decision-making processes, we can tap into their practical insights and expertise. This collaborative approach ensures that AMR mitigation strategies are not only scientifically sound but also feasible and effective within the agricultural context. However, it is important to acknowledge that reducing antibiotic use and implementing infection prevention measures can result in additional costs for farmers. These may include increased labour costs for more intensive management practices, improved animal housing, and enhanced biosecurity. These costs can be a concern for farmers as they may affect their economic sustainability. Additionally, reducing the use of antibiotics can potentially reduce the growth rate of livestock, leading to higher production costs.

These economic challenges can, in turn, affect food prices. As the cost of livestock production increases, this may lead to higher prices for animal-derived products. This has implications for consumers and can be a point of concern for policymakers.

Balancing the need for responsible antibiotic use and animal welfare with the economic sustainability of farmers and affordable food prices is a complex and ongoing challenge that requires collaboration between stakeholders, including farmers, veterinarians, policymakers, and consumers, to find sustainable and equitable solutions.

The One Health community comprises professionals from diverse disciplines, each with its unique culture, language, and conceptual framework. One of the significant challenges in addressing AMR within the One Health framework is the recognition that public health, animal health, and agroecosystem professionals often use similar terms, but they may define these terms differently. This disconnect in terminology and definitions can lead to misunderstandings and hinder effective collaboration between these disciplines. It is essential to bridge these gaps by fostering clear communication and shared understanding. Establishing common definitions and a unified language for discussing AMR-related concepts is crucial to facilitate cooperation and harmonize efforts across the One Health spectrum. Such collaboration is vital for successfully addressing the complex and interconnected issue of antibiotic resistance.

Furthermore, the antibiotic-resistant genes constituting the environmental ‘resistome’ get transferred to human and veterinary pathogens, a prevention strategy is needed that involves humans, animals, and the environment in a One Health perspective.

## 8. Conclusions

Addressing AMR comprehensively requires coordinated efforts to promote responsible antibiotic use, develop alternative therapies, enhance surveillance and monitoring, and implement policies that safeguard the effectiveness of existing antibiotics. The present review underscores the critical need for collaboration among all stakeholders engaged in the growth and management of AMR, underlining that achieving optimal health for people, animals, plants, and the environment requires a unified effort. This collaboration should involve stakeholders from various sectors, including human and veterinary medicine, agriculture, environmental science, policy making, and more. In addition, in addressing AMR from a One Health viewpoint, it is imperative to include an agricultural perspective. Farmers, ranchers, and agricultural professionals possess invaluable, firsthand knowledge of the intricate systems within their industry. They are the individuals on the front lines, responsible for implementing on-farm control measures to prevent AMR. 

By bringing together diverse expertise and perspectives, stakeholders can work towards solutions that protect public health, ensure food safety, sustain economic well-being, and preserve the health of ecosystems. The global nature of AMR necessitates a united front in the pursuit of One Health strategies to mitigate its impact and secure a healthier future for all.

## Figures and Tables

**Table 1:** Overview of the development of bacterial resistance against common antibiotics.

Antibiotic Class	Mechanism of Action	Examples of Antibiotics	Year Introduced to the Market	Year Antibiotic Resistance Identified
Penicillins (β-Lactam)	Inhibition of bacterial cell wall synthesis.	Penicillin	1943	1940, 1965, 1967, 1976
		Ampicillin	1961	1962, 1964
		Amoxicillin	1972	1977
		Methicillin	1960	1960
Cephalosporins (β-Lactam)		Cefotaxime	1980	1983
		Ceftaroline	2010	2011
	Avibactam inhibits serine β-lactamases enzyme	Ceftazidime (3rd Generation cephalosporins)	1984	1987
		Ceftazidime–avibactam	2015	2015
Aminoglycosides	Protein biosynthesis inhibition	Streptomycin	1944	1946
		Tobramycin	1967	1981
		Amikacin	1976	1981
		Gentamicin	1963	1973
		Neomycin	1952	1950
		Kanamycin	1957	1967
Chloramphenicol		Chloramphenicol	1948	1960
Glycopeptides	Cell wall synthesis inhibition	Vancomycin	1972	1988
		Teicoplanin (derivative of Vancomycin)	1984	1986
Ansamycins	RNA synthesis inhibition	Rifampin	1968	1972
Sulfonamides	DNA synthesis inhibition	Prontosil	1936, 1935	1942
		Sulfamethoxazole	1961	1960
Tetracyclines	Protein biosynthesis inhibition	Tetracycline	1950	1959
Macrolide		Erythromycin	1953	1956
		Azithromycin	1980	2011
Oxazolidinones		Linezolid	2000	2001
Quinolones	DNA synthesis inhibition	Ciprofloxacin	1987	2007
		Levofloxacin	1996	1996
Lipopeptides	Cell wall synthesis disruption disrupting multiple aspects of bacterial cell membrane function	Daptomycin	2003	2004
		Bacitracin	1945	1955
		Aztreonam	1984, 1986	1986
		Imipenem	1985	1996
Lincosamides	Interference with the synthesis of proteins	Clindamycin	1966	1971

## Data Availability

Data is contained within the article.
